# Cerebral imaging should also be performed to examine the visual pathways of people with diabetes using visually evoked potentials

**DOI:** 10.22336/rjo.2024.86

**Published:** 2024

**Authors:** Josef Finsterer

**Affiliations:** Neurology Department, Neurology & Neurophysiology Center, Vienna, Austria

**Keywords:** diabetes, retinopathy, visually-evoked potentials, polyneuropathy, visual impairment

**Letter to the Editor**

We were interested in reading the article by Ashok et al. on a prospective case-control study using pattern-reversal visual evoked potentials (VEPs) to assess the degree of visual pathway impairment in diabetic patients without retinopathy [1]. The P100 latency was prolonged, and the N75-P100 amplitude was reduced in diabetic patients [1]. In patients with diabetic polyneuropathy, the P100 latency was prolonged, and the N75-P100 amplitude was reduced compared to people with diabetes without polyneuropathy [1].

The P100 latency was positively correlated with the duration of diabetes, and the N75-P100 amplitude was negatively correlated with it [1]. It was concluded that VEP changes occur before the development of retinopathy or peripheral neuropathy [1]. The study is excellent, but some points should be discussed.

The first point is that cerebral imaging was not included in the study. The examination of the visual pathways using VEPs should also include the cerebral portion of the visual pathways, as prolongation of the P100 latency may not only be due to damage to the optic nerve or chiasm but also to damage to the retrochiasmatic portion of the visual pathways. Impairment of the retrochiasmatic visual pathways in diabetic patients may be due not only to diabetic encephalopathy but also to vascular dysfunction complicated by cerebral ischemia and stroke. Therefore, it is essential to combine VEPs with cerebral imaging to assess the function and morphology of the visual pathway. In this context, it is also vital that magnetic resonance imaging of the intra- and extracranial arteries and carotid ultrasound are performed to rule out narrowing or occlusion of any of the large or small arteries supplying the brain. Cerebral imaging is also required to rule out other causes of retro-retinal visual pathway impairment, such as mass lesions, hemorrhage, stroke, encephalitis, demyelination, or vasculitis. An adverse history of cerebral disease does not rule out the possibility that it was subclinical or occurred shortly before the VEP recordings.

The second point is that the study was conducted during the pandemic, so it is essential to rule out central nervous system involvement in the viral infection. We should know whether all included patients and controls were truly SARS-CoV-2 negative when recording the VEPs. Cerebral involvement in SARS-CoV-2 infection can be highly variable and can often only be confirmed by a comprehensive cerebrospinal fluid (CSF) examination. To rule out an infectious or immunological cause of visual impairment in diabetics, it would therefore have been imperative also to examine the CSF of those who tested positive for SARS-CoV-2.

Third, diabetic encephalopathy can manifest not only with visual impairment but also with cognitive impairment, so it would have been mandatory to test all included patients for cognitive impairment due to diabetic encephalopathy.

The fourth point is that polyneuropathy-associated diseases were not sufficiently excluded [1]. To exclude causes of polyneuropathy other than diabetes, it would have been imperative to screen all patients with neuropathy for alternative causes of polyneuropathy.

In summary, this interesting study has limitations that relativize the results and their interpretation. Addressing these limitations could strengthen the conclusions and corroborate the study’s findings. When examining the visual pathways in diabetic patients with visual evoked potentials, additional cerebral imaging should be performed to rule out other pathology.

## References

[1] Ashok K, Ankit P, Nitin PK, Sanjeev K (2024). Utility of Visual Evoked Potentials (VEPs) study in the evaluation of visual pathway dysfunction in people with diabetes without retinopathy: correlations with diabetic peripheral neuropathy and other clinical findings. Rom J Ophthalmol.

